# Evaluating the influence of a 3-min online video on the community knowledge of stroke in four Arab countries

**DOI:** 10.3389/fpubh.2024.1342490

**Published:** 2024-05-22

**Authors:** Katia Iskandar, Deema Rahme, Pascale Salameh, Chadia Haddad, Hala Sacre, Mohamed Bahlol, Rula M. Darwish, Sarah El Khatib, Jihan Safwan, Fouad Sakr, Mohamad Rahhal, Hassan Hosseini, Michelle Cherfane

**Affiliations:** ^1^School of Pharmacy, Lebanese International University, Beirut, Lebanon; ^2^INSPECT-LB (Institut National de Santé Publique, d’Épidémiologie Clinique et de Toxicologie-Liban), Beirut, Lebanon; ^3^Department of Health and Social Work, School of Public Health, Lebanese University, Fanar, Lebanon; ^4^Higher Institute of Public Health (ISSP), Saint Joseph University of Beirut, Beirut, Lebanon; ^5^Department of Pharmacy Practice, School of Pharmacy, Beirut Arab University, Beirut, Beirut, Lebanon; ^6^Department of Primary Care and Population Health, University of Nicosia Medical School, Nicosia, Cyprus; ^7^Gilbert and Rose-Marie Chagoury School of Medicine, Lebanese American University, Byblos, Lebanon; ^8^Faculty of Pharmacy, Lebanese University, Hadat, Lebanon; ^9^Research Department, Psychiatric Hospital of the Cross, Jal Eddib, Lebanon; ^10^Drug Information Center, Order of Pharmacists of Lebanon, Beirut, Egypt; ^11^Department of Pharmaceutical management and economics, Department Pharmacy Practice and Clinical Pharmacy, Faculty of Pharmacy, Egyptian Russian University, Badr city, Cairo governorate, Egypt; ^12^Department of Pharmaceutics and Pharmaceutical Technology, School of Pharmacy, The University of Jordan, Amman, Jordan; ^13^École Doctorale Sciences de la Vie et de la Santé, Université Paris-Est Créteil, Paris, France; ^14^UMR U955 INSERM, Institut Mondor de Recherche Biomédicale, Université Paris-Est Créteil, Paris, France; ^15^INSERM U955-E01, IMRB, Henri Mondor Hospital, Créteil, France; ^16^Department of Neurology, Henri Mondor Hospital, AP-HP, Créteil, France; ^17^Environmental and Public Health Department, College of Health Sciences, Abu Dhabi University, Abu Dhabi, United Arab Emirates

**Keywords:** stroke, knowledge, video, educational tool, Arab countries

## Abstract

**Introduction:**

Studies from developed and developing countries showed that the knowledge levels of stroke need improvement. Educational campaigns varied and were of limited influence predominantly because of their short duration and the need for financial support. The study aims to test the impact of a 3-min online video on the knowledge of stroke and factors influencing the knowledge score in four Arab countries.

**Methods:**

A cross-sectional web-based pre-post study was conducted in Egypt, Jordan, Lebanon, and the United Arab Emirates. The data were collected using the snowball technique. Participants were adults aged 18 years and above. The questionnaire sequence was conducting a pretest, followed by the educational video explaining stroke occurrence, types, risks, warning signs, preventive measures, and treatment, and finally, a posttest to evaluate the differences in knowledge from baseline. Statistical analysis included paired *t*-tests comparing pre-post-education stroke knowledge scores, while repeated measures ANOVA, adjusting for covariates, assessed mean changes.

**Results:**

The total number of participants was 2,721, mainly younger than 55 years. The majority had a university degree and were not healthcare professionals. A significant improvement was noted in the total knowledge score in all countries from a mean average (*M*_pretest_ = 21.11; *M*_posttest_ = 23.70) with *p* < 0.001. Identification of the stroke risks (*M*_pretest_ = 7.40; *M*_posttest_ = 8.75) and warning signs (*M*_pretest_ = 4.19; *M*_posttest_ = 4.94), understanding the preventive measures (*M*_pretest_ = 5.27; *M*_posttest_ = 5.39) and the importance of acting fast (*M*_pretest_ = 0.82; *M*_posttest_ = 0.85) improved from baseline with (*p* < 0.001) for all score components.

**Conclusion:**

The educational tool successfully enhanced public understanding of stroke risks, the identification of stroke signs, and the critical need for emergency action. The advantages of this video include its short length, free online access, use of evidence-based content in lay language, and reflective images. The ultimate goal remains the long-term improvement of sustainability by mandating full-scale trials.

## Introduction

Stroke is the second leading cause of morbidity and mortality worldwide (1). The number of deaths due to stroke was 6.6 million in 2020, a figure projected to increase by at least 50% by 2050 (1). The burden of disability linked with disease complications is also on the rise, with an estimated increase from 144.8 million disability-adjusted life-years (DALYs) in 2020 to 189.3 million DALYs by 2050 (1). Previous data on the incidence and deaths due to stroke have been markedly and rapidly increasing since the 1990s, predominantly in low- and middle-income countries (LMICs) (1). The recent alarming findings are the higher incidence of stroke in young individuals (18–50 years) (2, 3). Stroke is also associated with high economic impact, with estimated costs of treatment, rehabilitation, and loss of productivity exceeding US$891 billion per year globally (1). Evidence-based solutions have been proposed to reduce the global incidence of stroke based on the following four pillars: surveillance, prevention, acute care, and rehabilitation (1). Primordial prevention of stroke meets the sustainable development goals (4), including (1) reducing poverty, (2) improving socioeconomic conditions, (3) universal health coverage, (4) health equity, (5) reducing air pollution, (6) healthy lifestyle, (7) building healthy cities and homes, (8) and public health campaigns to raise awareness about stroke and stroke risk factors (1).

The World Health Organization and the Lancet Neurology Commission surveyed 84 LMICs and high-income countries (HICs) to compare the status of stroke services. The results showed numerous deficiencies, including a lack of public education in many countries (5). The scope of this study is primordial community education as a preventive measure of stroke occurrence and recurrence (6). Primordial stroke prevention consists of activities to avoid stroke risk factors, whereas primary prevention aims to limit or manage exposure to risk factors for stroke (6). Research showed that the community knowledge of stroke symptoms, prevention measures, and the urgency of hospitalization in case of an event is poor or lacking in both LMICs and HICs (7–9).

Educational campaigns, including mass media (10), and digital platforms such as mobile applications [e.g., the PreventS-MD web app (PreventS-MD) (11) and stroke riskometer (12)], text messages to mobile health telemedicine (12), computer and web-based technologies, including artificial intelligence, are adapted tools to support stroke knowledge, awareness, and management (6). A systematic review and meta-analysis found that public stroke campaigns significantly influence the identification of stroke symptoms and the urgency for hospital transfer (13). The study stressed the accessibility and affordability of the digital technologies used by lay individuals for primary stroke prevention and considered that evidence-based content improves physician–patient communication and healthcare provision (13). Educating young adults about stroke was found beneficial in spreading awareness among families, raising the alarms about harmful health behaviors, and adapting preventive measures to reduce the burden of stroke in adult life (14). Numerous studies have demonstrated the effectiveness of educational campaigns and other digital tools in enhancing public knowledge of stroke to modify health behaviors and minimize delays in emergency transfer of a patient experiencing stroke (15–17). In this study, a 3-min educational video was built based on the American Stroke Association online awareness materials (18), the BE-FAST algorithm (19), and the Stroke Foundation New Zealand (20). The BE-FAST algorithm allows the identification of persons experiencing an ischemic stroke episode (18) to act fast and avoid delays in seeking emergency care. This study aims to test the impact of the budget-free short educational online video on public knowledge in Arab countries about stroke and factors influencing stroke knowledge scores.

## Materials and methods

### Study design

A cross-sectional population-based interventional study was conducted from 1 July 2022 to September 2022. The research proposal was sent to international research groups in Arab countries to invite interested parties to join the study. Researchers from Egypt, Jordan, Lebanon, and the United Arab Emirates (UAE) agreed to join the project. Google Forms, a cloud-based survey powered by Google, was used to create the online survey, which included a 3-min educational video based on the BE-FAST algorithm (19). Data were collected using the snowball sampling technique (21) by asking university students to share the link to the online survey with their families and social networks using various social media platforms (WhatsApp, Facebook, Instagram, and LinkedIn).

### Participants

Eligible participants were adults 18 years of age or older, with internet access, and residing in the participating countries. The first contact with participants was made through university students in their respective countries who were trained and informed about the content and aim of the survey. The total number of respondents from the four participating countries was 2,721, including 1,414 from Egypt, 215 from Jordan, 685 from Lebanon, and 407 from the UAE.

### Sample size calculation

The minimum sample size was calculated using the G-Power software, version 3.0.10. Taking into consideration that a stratified analysis per country applies, the minimum sample size calculation was determined as follows: the calculated effect size was 0.11 (small effect size), based on an expected squared multiple correlation of 0.1 (R2 deviation from 0) related to the omnibus test of multiple regression. The minimum sample required was n = 205 from each country, considering an alpha error of 5%, a power of 80%, and allowing 20 predictors in the model.

### Questionnaire/educational video

The video was 3 min in length, and the evidence-based content was presented in lay language with images reflective of the content. The online video was piloted among the researcher’s network to test the acceptance and understanding of the content. Expert opinions of healthcare professionals provided insights about the relevance of the content. The purpose of the questionnaire/video was to educate participants about stroke, including its occurrence, risk factors, alarming signs, treatment, and prevention measures. Pretests and posttests included the same questions to evaluate baseline participant knowledge about the material in the video and evaluate acquired knowledge post-video. The online survey is translated from English to Arabic according to the WHO translational guidelines^1^. The translated version was then back-translated to English again. English versions were compared, with minor discrepancies corrected by consensus between the translators and the principal investigator in each country.

The questionnaire consisted of three main parts sequenced as follows: pretest, 3-min educational video, and posttest. It included closed-ended questions and encompassed the following four sections:

1. Participants’ sociodemographic characteristics, health status, and lifestyle. A healthy lifestyle index was set as any individual with normal-range body mass index (BMI), exercising regularly (mild, moderate, or intense), non-smoker or currently non-smoker, not drinking alcohol, eating fruits and vegetables, and drinking enough fluids (22).
2. A pretest that assesses the respondent’s baseline knowledge of stroke: (a) occurrence site, (b) risk factors (i.e., uncontrolled hypertension, diabetes, arrhythmias, dyslipidemia, transient ischemic attack, previous or family history of stroke, stress, obesity, smoking, alcohol consumption, aging, and poor diet), (c) alarming signs (i.e., sudden onset of weakness or numbness on one side of the body, sudden speech difficulty or confusion, sudden difficulty seeing in one or both eyes, and sudden severe headache with unknown cause), (d) preventive measures (i.e., keeping a healthy body weight, quit smoking and stop drinking alcohol, regular exercise, eating a healthy diet, reducing stress, and regular use of prescribed medications to treat underlying diabetes and cardiovascular diseases, including control of blood pressure), and (e) calling the ambulance. The pretest questions were based on the risks and warning signs established by the American Stroke Association (18) and the National Institute of Neurological Disorders and Stroke (23).
3. A 3-min online educational video: “Think and Act Fast to Save a Life” (English Version: https://youtu.be/uzN-Q5SUKmA; Arabic Version: https://youtu.be/yEvvQlX37gs).
4. A posttest, including the same set of questions as the pretest.

### Reliability and validity testing of the questionnaire

A factor analysis was conducted on responses from 250 participants to explore stroke knowledge. The Kaiser–Meyer–Olkin measure indicated excellent sampling adequacy (KMO = 0.960), and Bartlett’s test supported the factorability of the correlation matrix (χ^2^ = 1502.45, df = 300, *p* < 0.001). Principal component analysis with Promax rotation extracted four factors, explaining 56.74% of the variance. Factors included prevention measures, risk of stroke, warning signs and treatment, and general knowledge and type of stroke. Reliability analysis yielded Cronbach’s alpha of 0.945. The scales used were of appropriate validity and reliability (Table 1).

**Table 1:** Factor analysis of the knowledge of stroke (Promax rotated component matrix).

	Factor 1	Factor 2	Factor 3	Factor 4
Exercise regularly	0.899			
Follow-up with your doctor	0.889			
Avoid stress	0.875			
Maintain a healthy weight	0.873			
Eat healthy food	0.863			
Take your medications on time	0.838			
The risk of stroke increases when you have heart diseases		0.881		
The risk of stroke increases when having diabetes mellitus		0.847		
The risk of stroke increases when obesity		0.810		
The risk of stroke increases when you have irregular heart rhythm		0.777		
The risk of stroke increases when you smoke		0.654		
The risk of stroke increases when drinking alcohol		0.580		
The risk of stroke increases when you do not engage in regular exercise		0.564		
The risk of stroke increases when you have uncontrolled BP		0.421		
The risk of stroke increases when you are stressed		0.284		
The risk of stroke increases if you are a male		0.288		
The risk of stroke increases if you are 55 years and older		0.195		
Eye falling downward			0.827	
Not being able to answer questions			0.789	
Dizziness			0.741	
Severe headache			0.720	
Unable to speak properly			0.691	
Paralysis in the arm or leg or both			0.644	
How stroke is treated by rehabilitation			0.503	
How stroke is treated by medications			0.340	
Stroke is a serious condition that occurs in the brain				0.892
Stroke occurs when the arteries in the brain are partially or completely blocked				0.859
Stroke occurs when the brain arteries bleed				0.551
Call an ambulance straight away				0.292

Factor 1: prevention measure; Factor 2: Risk of stroke; Factor 3: warning signs and treatment of stroke; Factor 4: general knowledge and type of stroke. Kaiser–Meyer–Olkin (KMO) 0.960, Bartlett’s test of sphericity < 0.001, Percentage of variance explained 56.74%.Cronbach’s alpha value: 0.945.

### Ethical approval

The Institutional Review Board at Abu Dhabi University approved this study under the code number CoHS – 22-05-00018 on 16 May 2022. The study complies with the Declaration of Helsinki (24). Before filling out the online survey, participants were informed about the study objectives and the freedom to withdraw at any time. They also were informed that their participation is anonymous and voluntary. All participants filled out informed consent included at the beginning of the survey forms to enable proceeding with the survey. Participants did not receive any financial reward in exchange for their participation.

### Statistical analysis

Data were analyzed using SPSS software version 25. The descriptive analysis used absolute frequencies and percentages for categorical variables and means and standard deviations (SD) for quantitative measures. As the skewness and kurtosis values of the dependent variables (knowledge total score) were under the acceptable range − 2 and + 2, so the data were considered normally distributed.

The paired sample *t*-test compared the stroke knowledge score before and after the education session. A repeated measures ANOVA evaluated the mean change of the stroke total knowledge before and after the educational video tool adjusted for covariates (age, gender, marital status, smoking, alcohol, education level, healthy lifestyle, having a stroke, family history of stroke, and having any medical illness). A bivariate analysis was conducted, taking the knowledge total score (pre, post, and the difference) as the dependent variables. Student’s *T*-test and ANOVA test were used in the bivariate analysis to compare two or more than three means. Cohen’s d effect size was calculated using the Psychometrica tool^2^. According to the effect size classification, a small effect was found for d = 0.2, medium d = 0.5, and large d ≥ 0.8. The *post-hoc* power was calculated using the G Power software. A *p-value* < 0.05 was considered significant.

## Results

### Demographic characteristics

A total of 2,721 participants were enrolled in the study from four Arab countries (Lebanon, UAE, Jordan, and Egypt). In Egypt and the UAE, sex-disaggregated responses are more balanced (approximately 50% women) than in Jordan and Lebanon, where the majority of respondents were women (≥63%). Nearly half or more of the participants were young, aged between 25 and 34 years, and almost all were highly educated (≥88%) but predominantly not healthcare providers (>66%) (Table 2).

**Table 2:** Demographic characteristics of the participants.

*N* = 2,721				
Variable	Lebanon (*N* = 685)	UAE (*N* = 407)	Jordan (*N* = 215)	Egypt (*N* = 1,414)
**Gender**				
Male	248 (36.2%)	189 (46.4%)	79 (36.7%)	653 (46.2%)
Female	437 (63.8%)	218 (53.6%)	136 (63.3%)	761 (53.8%)
**Age in years**				
25–34	285 (41.6%)	207 (50.9%)	123 (57.2%)	929 (65.7%)
35–44	127 (18.5%)	110 (27.0%)	21 (9.8%)	246 (17.4%)
45–54	155 (22.6%)	65 (16.0%)	40 (18.6%)	184 (13.0%)
55 and above	118 (17.2%)	25 (6.1%)	31 (14.4%)	55 (3.9%)
**Marital status**				
Single/widowed/divorced	325 (47.4%)	180 (44.2%)	131 (60.9%)	857 (60.6%)
Married	360 (52.6%)	227 (55.8%)	84 (39.1%)	557 (39.4%)
**Education level**				
Primary	30 (4.4%)	11 (2.7%)	7 (3.3%)	24 (1.7%)
Secondary	50 (7.3%)	38 (9.3%)	17 (7.9%)	28 (2.0%)
University	605 (88.3%)	358 (88%)	191 (88.8%)	1,362 (96.3%)
**Being a healthcare professional**				
Yes	135 (19.7%)	104 (25.6%)	72 (33.5%)	436 (30.8%)
No	550 (80.3%)	303 (74.4%)	143 (66.5%)	978 (69.2%)

UAE, United Arab Emirates.

### Risks of stroke among participants

The majority of respondents were not at risk of developing stroke. They did not have a family history of stroke (>64%) or a previous stroke history (>96%). The majority of the participants were non-smokers and did not consume alcohol except 46.3% in Lebanon versus more than 84.8% in other countries. Nearly half of the people surveyed engage in mild physical activity and are either overweight or obese. Participants most frequently reported experiencing severe headaches, elevated cholesterol, and a family history of myocardial infarction (Table 3).

**Table 3:** Stroke risk factors among participants.

Variable	Lebanon (*N* = 685)	UAE (*N* = 407)	Jordan (*N* = 215)	Egypt (*N* = 1,414)
**Family history of stroke**				
Yes	244 (35.6%)	126 (31.0%)	68 (31.6%)	284 (20.1%)
No	441 (64.4%)	281 (69.0%)	147 (68.4%)	1,130 (79.9%)
**Previous stroke**				
Yes	14 (2.0%)	13 (3.2%)	6 (2.8%)	57 (4.0%)
No	671 (98.0%)	394 (96.8%)	209 (97.2%)	1,357 (96.0%)
**Smoking**				
Never smoke	401 (58.5%)	276 (67.8%)	151 (70.2%)	1,245 (88.0%)
Current smoker	234 (34.2%)	104 (25.6%)	49 (22.8%)	115 (8.1%)
Former smoker	50 (7.3%)	27 (6.6%)	15 (7.0%)	54 (3.8%)
**Alcohol**				
Do not drink	317 (46.3%)	345(84.8%)	212 (98.6%)	1,348 (95.3%)
Occasionally	261 (38.1%)	55 (13.5%)	2 (0.9%)	26 (1.8%)
On weekends	83 (12.1%)	7 (1.7%)	1 (0.5%)	15 (1.1%)
Daily	24 (3.5%)	–	–	25 (1.8%)
**Physical activity**				
Not applicable	24 (3.5%)	24 (5.9%)	3 (1.4%)	76 (5.4%)
Mild	359 (52.4%)	215 (52.8%)	138 (64.2%)	774 (54.7%)
Moderate	229 (33.4%)	124 (30.5%)	63 (29.3%)	460 (32.5%)
Vigorous	73 (10.7%)	44 (10.8%)	11 (5.1%)	104 (7.4%)
**BMI**				
Underweight	24 (3.5%)	14 (3.4%)	12 (5.6%)	34 (2.4%)
Healthy weight	334 (48.8%)	179 (44.0%)	101 (47.0%)	587 (41.6%)
Overweight	225 (32.8%)	134 (32.9%)	67 (31.2%)	505 (35.8%)
Obese	102 (14.9%)	80 (19.7%)	35 (16.3%)	286 (20.3%)
**Having any of the following health conditions**				
High blood pressure	110 (16.1%)	70 (17.2%)	36 (16.8%)	273 (19.4%)
History of MI	35 (5.1%)	17 (4.2%)	5 (2.3%)	69 (4.9%)
Family history of MI	184 (26.9%)	50 (12.3%)	36 (16.9%)	198 (14.1%)
History of CAD	65 (9.5%)	27 (6.6%)	10 (4.7%)	99 (7.0%)
High cholesterol level	130 (19.0%)	71 (17.5%)	36 (16.7%)	233 (16.6%)
Diabetes type 2	68 (9.9%)	56 (13.8%)	19 (8.8%)	155 (11.0%)
History of DVT	30 (4.4%)	14 (3.4%)	11 (5.1%)	81 (5.7%)
History of PE	19 (2.8%)	13 (3.2%)	8 (3.7%)	70 (5.0%)
Severe headache (migraine)	132 (19.3%)	90 (22.3%)	33 (15.3%)	407 (29.2%)

CAD, coronary artery disease; DVT, deep vein thrombosis; Former smoker (i.e., quit more than 6 months ago); MI, myocardial infarction; PE, pulmonary embolism; UAE, United Arab Emirates; BMI, Body Mass Index.

### Comparison of stroke knowledge pretest and posttest

A significant increase was found post-education in the total knowledge score and among all knowledge items (*p* < 0.001), including stroke site of occurrence, types, risks, warning signs, prevention measures, treatment, and acting fast to save life (Table 4).

**Table 4:** Variation of the stroke knowledge pre- and post-education session.

	Pre	Post	Effect size d_Cohen_	95% CI of effect size	*Post hoc* power	*p*-value
	Mean ± SD	Mean ± SD				
Knowledge total score	21.11 ± 7.78	23.70 ± 7.51	0.34	0.26; 0.41	1.00	<0.001
Stroke is a serious condition that occurs in the brain	0.83 ± 0.37	0.85 ± 0.34	0.06	−0.02; 0.13	0.87	<0.001
**Types of strokes**						
Stroke occurs when the arteries in the brain are partially or completely blocked	0.74 ± 0.43	0.82 ± 0.38	0.20	0.12; 0.27	1.00	<0.001
Stroke occurs when the brain arteries bleed	0.50 ± 0.50	0.68 ± 0.46	0.37	0.30; 0.45	1.00	<0.001
**Risk of stroke**	7.40 ± 3.50	8.65 ± 3.22	0.37	0.30; 0.45	1.00	<0.001
The risk of stroke increases when you have uncontrolled BP	0.77 ± 0.41	0.86 ± 0.34	0.24	0.16; 0.31	1.00	<0.001
The risk of stroke increases when you have irregular heart rhythm	0.59 ± 0.49	0.74 ± 0.43	0.32	0.25; 0.40	1.00	<0.001
The risk of stroke increases when obesity	0.62 ± 0.48	0.75 ± 0.43	0.28	0.21; 0.36	1.00	<0.001
The risk of stroke increases when having diabetes mellitus	0.54 ± 0.49	0.70 ± 0.45	0.34	0.26; 0.42	1.00	<0.001
The risk of stroke increases when you have heart diseases	0.64 ± 0.47	0.74 ± 0.43	0.22	0.15; 0.30	1.00	<0.001
The risk of stroke increases when you smoke	0.78 ± 0.41	0.86 ± 0.34	0.21	0.14; 0.28	1.00	<0.001
The risk of stroke increases when drinking alcohol	0.74 ± 0.43	0.83 ± 0.36	0.23	0.15; 0.30	1.00	<0.001
The risk of stroke increases when you do not engage in regular exercise	0.72 ± 0.44	0.84 ± 0.36	0.30	0.22; 0.37	1.00	<0.001
The risk of stroke increases when you are stressed	0.81 ± 0.38	0.86 ± 0.34	0.14	0.06; 0.21	1.00	<0.001
The risk of stroke increases if you are a male	0.44 ± 0.49	0.64 ± 0.47	0.42	0.34; 0.49	1.00	<0.001
The risk of stroke increases if you are 55 years and older	0.71 ± 0.45	0.79 ± 0.40	0.20	0.11; 0.26	1.00	<0.001
**Warning signs**	4.19 ± 2.10	4.84 ± 1.89	0.32	0.25; 0.40	1.00	<0.001
Unable to speak properly	0.78 ± 0.41	0.86 ± 0.34	0.21	0.14; 0.28	1.00	<0.001
Eye falling downward	0.62 ± 0.48	0.75 ± 0.43	0.28	0.21; 0.36	1.00	<0.001
Paralysis in the arm or leg or both	0.72 ± 0.44	0.80 ± 0.39	0.19	0.12; 0.27	1.00	<0.001
Dizziness	0.66 ± 0.47	0.79 ± 0.40	0.30	0.22; 0.37	1.00	<0.001
Severe headache	0.69 ± 0.45	0.81 ± 0.38	0.29	0.21; 0.36	1.00	<0.001
Not being able to answer questions	0.71 ± 0.45	0.81 ± 0.38	0.24	0.16; 0.32	1.00	<0.001
**Prevention measures**	5.27 ± 1.68	5.39 ± 1.60	0.07	−0.002; 0.15	0.95	<0.001
Eat healthy food	0.89 ± 0.30	0.91 ± 0.28	0.07	−0.006; 0.14	0.95	<0.001
Exercise regularly	0.87 ± 0.33	0.89 ± 0.30	0.06	−0.01; 0.14	0.87	<0.001
Avoid stress	0.88 ± 0.32	0.90 ± 0.30	0.06	−0.01;0.14	0.87	0.002
Maintain a healthy weight	0.85 ± 0.35	0.88 ± 0.31	0.09	0.02; 0.16	0.99	<0.001
Take your medications on time	0.88 ± 0.32	0.89 ± 0.30	0.03	−0.04; 0.10	0.34	<0.001
Follow-up with your doctor	0.88 ± 0.31	0.90 ± 0.29	0.07	−0.01; 0.14	0.95	0.007
**Treatment of stroke**						
How stroke is treated by medications	0.74 ± 0.43	0.85 ± 0.34	0.28	0.21; 0.36	1.00	<0.001
How stroke is treated by rehabilitation	0.58 ± 0.49	0.72 ± 0.44	0.30	0.22; 0.38	1.00	<0.001
**Acting fast in the case of stroke**						
Call an ambulance straight away	0.82 ± 0.37	0.85 ± 0.36	0.08	0.01; 0.16	0.98	<0.001

**Figure 1:**
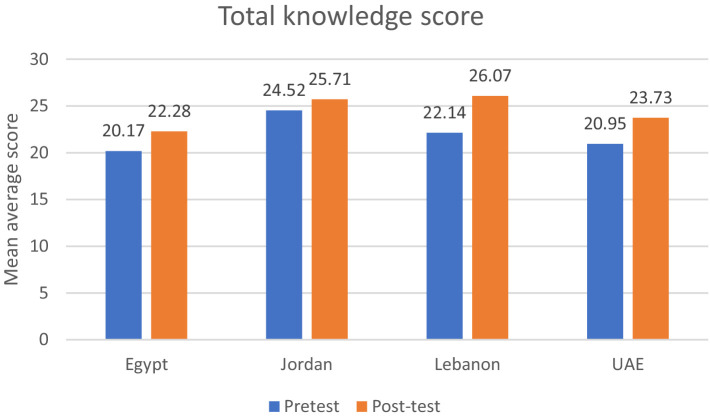
Differences in total knowledge pre-education and post-education scores among participating countries.

Figure 1 shows the means of the knowledge total score before and after the educational session, after adjustment over age, gender, marital status, smoking, alcohol, education level, healthy lifestyle, having a stroke, a family history of stroke, and any medical illness. A significantly higher increase in the total knowledge score was found post-education in all countries (*p* < 0.05).

### Bivariate analysis

The highest baseline mean knowledge score about stroke was found in Jordan (24.84 ± 4.55), followed by Lebanon (*M* = 22.26; SD = 6.22). The difference between the pre- and posttest was significant in all countries (*p* < 0.001). Although women had a higher baseline score (*M* = 21.67; SD = 7.15), the differences between pre and posttest were not significant for either gender (*p* = 0.353). The youngest participants (25–34 years) had a significantly lower mean knowledge score at baseline (*M* = 20.74; SD = 8.18) than participants 55 years and older (22.46 ± 6.00). There was no significant difference in the mean knowledge score between participants with and without a university degree, although highly educated participants had a higher baseline score (*p* = 0.425). Results showed that individuals who consumed alcohol had higher mean score differences (*M* = 3.12; SD = 5.21), while those with a low healthy lifestyle index had significantly higher knowledge scores (*M* = 2.68; SD = 5.40) (Table 5).

**Table 5:** Factors affecting the knowledge score.

Variable	Knowledge score											
	Pretest	*p-value*	Effect Size d_Cohen_ (95% CI)	*Post hoc* power	Posttest	*p-value*	Effect size d_Cohen_ (95% CI)	*Post hoc* power	Difference Post-pre test	*P value*	Effect Size d_Cohen_ (95% CI)	*Post hoc* power
					Mean (SD)							
Country												
Lebanon	22.26 ± 6.22	<0.001	0.57 (0.45; 0.74)	1.00	26.06 ± 4.95	<0.001	0.46 (0.43; 0.61)	1.00	3.80 ± 4.92	<0.001	0.68 (0.39; 0.70)	1.00
UAE	20.80 ± 8.58				23.53 ± 8.16				2.72 ± 5.60			
Jordan	24.84 ± 4.55				26.07 ± 5.13				1.23 ± 3.77			
Egypt	20.08 ± 8.34				22.25 ± 8.24				2.16 ± 5.54			
Gender												
Male	20.37 ± 8.48	<0.001	0.17 (0.09; 0.24)	0.99	22.85 ± 8.45	<0.001	0.20 (0.12; 0.27)	0.99	2.47 ± 5.60	0.353	0.04 (−0.04; 0.11)	0.18
Female	21.67 ± 7.15				24.34 ± 6.65				2.67 ± 5.13			
Age (years)												
25–34	20.74 ± 8.18	0.006	0.21 (0.08; 0.36)	1.00	22.98 ± 8.18	<0.001	0.33 (0.21; 0.48)	1.00	2.24 ± 5.33	<0.001	0.19 (0.05; 0.33)	1.00
35–44	21.18 ± 7.75				23.90 ± 7.21				2.71 ± 5.61			
45–54	21.63 ± 7.04				24.94 ± 5.98				3.30 ± 4.98			
55 and above	22.46 ± 6.00				25.69 ± 5.11				3.23 ± 5.22			
Marital status												
Single/widowed/divorced	20.88 ± 8.03	0.083	0.07 (−0.01; 0.14)	0.44	23.21 ± 8.02	<0.001	0.14 (0.07; 0.22)	0.95	2.33 ± 5.42	0.006	0.10 (0.03; 0.18)	0.73
Married	21.40 ± 7.44				24.29 ± 6.80				2.89 ± 5.21			
University degree												
No	19.58 ± 8.67	0.008	0.21 (0.07; 0.35)	0.82	22.45 ± 9.12	0.040	0.18 (0.04; 0.32)	0.69	2.87 ± 5.02	0.425	−0.06 (−0.20; 0.08)	0.13
Yes	21.24 ± 7.69				23.80 ± 7.36				2.56 ± 5.36			
Being a healthcare professional												
No	20.46 ± 7.78	<0.001	0.31 (0.22; 0.39)	0.99	23.53 ± 7.52	0.054	0.08 (−0.002; 0.17)	0.46	3.06 ± 5.59	<0.001	−0.33 (−0.42; −0.25)	1.00
Yes	22.83 ± 7.50				24.15 ± 7.47				1.31 ± 4.35			
Previous stroke												
No	21.18 ± 7.71	0.054	−0.25 (−0.46; −0.04)	0.64	23.82 ± 7.40	0.001	−0.47 (−0.68; −0.26)	0.99	2.63 ± 5.35	0.006	−0.29 (−0.50; −0.08)	0.77
Yes	19.24 ± 9.31				20.31 ± 9.66				1.06 ± 4.70			
Family history of stroke												
No	20.55 ± 8.04	<0.001	0.28 (0.19; 0.36)	0.99	23.31 ± 7.82	<0.001	0.20 (0.11; 0.28)	0.99	2.75 ± 5.55	0.003	−0.12 (−0.20; −0.03)	0.78
Yes	22.67 ± 6.75				24.78 ± 6.47				2.11 ± 4.66			
Any high-risk disease												
No	21.06 ± 8.13	0.628	0.02 (−0.05; 0.09)	0.08	23.57 ± 7.91	0.248	0.04 (−0.03; 0.12)	0.18	2.50 ± 5.55	0.359	0.04 (−0.04; 0.11)	0.18
Yes	21.21 ± 7.34				23.90 ± 7.00				2.69 ± 5.06			
Current smoker												
No	21.13 ± 7.88	0.819	−0.01 (−0.11; 0.08)	0.05	23.69 ± 7.57	0.835	0.01 (−0.08; 0.11)	0.05	2.55 ± 5.33	0.540	0.03 (−0.07; 0.13)	0.09
Yes	21.04 ± 7.32				23.76 ± 7.28				2.71 ± 5.33			
Consumes alcohol												
No	21.04 ± 7.89	0.199	0.06 (−0.04; 0.17)	0.20	23.52 ± 7.61	0.002	0.15 (0.05; 0.25)	0.80	2.48 ± 5.35	0.023	0.12 (0.02; 0.22)	0.62
Yes	21.53 ± 7.14				24.66 ± 6.91				3.12 ± 5.21			
Healthy lifestyle index												
No	20.87 ± 7.75	<0.001	0.21 (0.11; 0.32)	0.97	23.55 ± 7.58	0.007	0.14 (0.03; 0.24)	0.74	2.68 ± 5.40	0.022	−0.12 (−0.22; −0.01)	0.61
Yes	22.50 ± 7.70				24.57 ± 6.99				2.06 ± 4.93			

Knowledge score is equal to the total knowledge scores of stroke occurrence, types, risks, warning signs, preventive measures, and what to do in case of an acute event.

## Discussion

In this study, the educational video successfully improved stroke knowledge among highly educated participants from different age groups and Arab countries. The predominant originality includes its brevity (which maintains audience focus), evidence-based content (18–20, 23), and availability online free of charge in English and Arabic. The educational tool effectively delivered relevant information about stroke occurrence sites, types, risk factors, warnings, signs, preventive measures, and appropriate behavior in emergencies, as evidenced by the significant improvement in all knowledge scores. The use of lay language and suggestive images made the content easily understood by the public and healthcare professionals. Improving knowledge, especially at a young age, is crucial to raising awareness of inappropriate health behaviors that may increase the risk of stroke. It could also help save lives by identifying alarming signs in case of an event and understanding the importance of seeking emergency care.

The pretest knowledge scores varied across countries, with Jordan exhibiting the highest scores, followed by Lebanon, while Egypt and UAE had comparable knowledge. Previous research in Jordan had categorized the level of knowledge and awareness regarding stroke as good, particularly among those with a higher level of education (25). Conversely, studies conducted in Lebanon have reported poor knowledge in the general population, but not among participants with a university degree (26), a trend also observed among older adults (24). Similarly, in the present study, 88.3% of the Lebanese participants had a university degree. Unfortunately, campaigns intended to educate the general population are limited and lacking in Lebanon. Public education about stroke currently relies on academia and the scientific communities’ efforts (27, 28). Since awareness campaigns need general budgeting and planning, the main barriers in Lebanon are the ongoing economic downturn, the health system collapse, and the political crisis (29). In Egypt, a low level of stroke knowledge was reported across four governorates (30), with participants from urban areas demonstrating better knowledge than their rural counterparts (31). Egypt and the UAE have established strategies to manage, prevent, and improve awareness of stroke (32–34) (35–39). In Egypt, accredited stroke centers, including tele-stroke units and training and education centers, have been established in collaboration with the Wings of Angels initiative (32, 40). While the UAE is engaged in multiple awareness campaigns about stroke and is invested in stroke management and prevention plans through partnerships (33) and the implementation of centers for excellence (34), stroke knowledge was better among educated individuals than the general public (41).

The baseline knowledge of stroke varied across different age groups, with participants aged 55 years and above scoring the highest (*M* = 22.46; SD = 6.00) compared to those aged 25–34 years (*M* = 20.74; SD = 8.18). According to the World Health Organization (WHO), stroke is the second leading cause of death in individuals aged >60 years and the fifth in people aged 15–59 years (42). The youngest age group (25–34 years) may not be aware of the high incidence of stroke reported at this age, which can explain the lower scores compared with older adults. In the last decade, stroke has been increasingly documented in young adults (1, 2, 42), potentially due to higher incidences of preventable risk factors, such as smoking, obesity, hypertension, and dyslipidemia (2, 4). Therefore, there is a critical need for educating young adults about the risks associated with stroke and the importance of preventive measures.

The baseline knowledge scores were lower among non-healthcare professionals, participants who did not have a family history of stroke, and those who consumed alcohol or did not maintain a healthy lifestyle. Interestingly, the difference between the pretest and posttest scores was significantly higher. These results indicate that the 3-min educational video succeeded in capturing the attention of susceptible individuals and those who may not have prior knowledge of this topic.

Educational campaigns have consistently demonstrated a significant improvement in stroke knowledge, awareness, and appropriate emergency response behaviors (14, 15, 43). In addition to educational lectures (44), these campaigns have used various tools, including mass media (18–20, 44–46), social media platforms (44, 47), mobile applications (14, 15), pharmacy flyers (47), readings in schools and high schools (44), and advertisement in public transportation spots (48). Notably, mobile applications were well-received by healthcare professionals and individuals at risk of stroke (14, 15). In Western Norway, mass media intervention effectively enhanced awareness about acute stroke onset, as indicated by the increase in the number of admissions to the emergency room (35). However, sustaining this improvement necessitated repeated campaigns beyond the initial 6 months allocated (35). Similarly, regular mass media coverage was recommended in Hungary to educate the public about stroke instead of a 1-day campaign (36). The Stroke Foundation in Australia conducted annual paid advertisements from 2004 to 2014 using the FAST campaign to promote the need for emergency medical services, resulting in improved public awareness and behaviors (37). Similar positive effects were observed in New Zealand with the implementation of these campaigns (38). Worldwide national campaigns among the general public significantly improved knowledge of stroke (39, 43, 45, 46) and emergency response behaviors (10, 43). These campaigns varied in duration, from 1-week campaigns per year for 4 years to 50-month initiatives (31).

The design and implementation of educational campaigns for stroke vary based on the sustainability of financial support and the intended audience’s demographics, including age, sex, cultural background, level of education, and stroke risks (43, 48–51). Reading was the preferred way to acquire information in schools (44). In some cultures, social networking may be a way of communicating and enhancing awareness about the topic (49). Television remains a widely accepted and efficient means of education and communication, reaching a vast audience. Nevertheless, it is time-limited and requires repetition to convey the message and a dedicated budget for sustained campaigns (44, 45, 50, 51). Social media platforms offer enhanced communication and interaction among healthcare professionals and the community (47). Blogs, infographics, and videos shared on social media can reach a vast audience (47). However, the proliferation of infodemics and misinformation on social media can have adverse effects on individuals’ health-related behaviors (52, 53).

Finally, the main challenges in stroke education campaigns include the need for short-term non-repetitive campaigns, sustainable financial support, and an adequate educational approach tailored to the cultural background, age, sex, and level of education of the target audience. Educating children in schools and colleges is crucial to preparing a knowledgeable society for the future and influencing the parents’ behaviors. Engaging students in academic activities allows them to undertake periodic educational campaigns within their community as part of extra-curricular public health initiatives. This bundle of initiatives may be effective even without a budget or financial support.

### Limitations

The limitations of the study are mainly the population distribution. Participants had predominantly a university degree, were 55 years and younger, and were women. Other limitations inherent to the study design include response bias and concerns related to data validity, as participants can get the information from another source, so the presence of an interviewer is preferable. The study design can also introduce selection bias, addressing the need to conduct randomized controlled trials to allow the generalizability of data findings. In addition, a comparison between intervention and control groups was not undertaken due to the study design limitations, lack of funding, and time constraints. Additional limitations include the short-term impact of this study, while detection of the tool’s long-term effectiveness in reducing disability, morbidity, and mortality and improving overall outcomes of stroke mandate a full-scale trial. Other limitations include a lack of long-term follow-up to verify the sustainability of the knowledge gained about stroke follow-up.

### Practical implications

The educational video in this study showed how academia can play a crucial role in public health education without the need for a high budget. There is room for improvement if the government, professional societies, healthcare professionals, social workers, academia, students, and the community engage in awareness campaigns. If a budget is available, television and advertisements in public transportation, flyers distribution in pharmacies, stroke screening, and educational campaigns have proven effective. Regardless of budget constraints, school education and the role of academia, professional societies, and international organizations can secure a sustainable impact for a healthy society.

## Conclusion

Educating the public about stroke has proven effective in enhancing knowledge and adapting appropriate behavior in case of an emergency. The main goal of this educational video in the short term is to improve community knowledge about stroke and the impact of inappropriate health behaviors, the identification of stroke signs, and action taken in case of an event. The mainstays are predominantly the sustainability and long-term effectiveness of this improvement in modifying inappropriate health behaviors and decreasing the risk of stroke morbidity and mortality. There is a need to capitalize on the strengths of this video and tailor its content to different cultural and educational backgrounds to reach all age groups. This tool can be part of a bundle of evidence-based interventions planned and implemented in collaboration with healthcare providers, the academic sector, governments, non-healthcare providers, and community engagement.

## Data availability statement

The datasets presented in this study can be found in online repositories. The names of the repository/repositories and accession number(s) can be found at: https://doi.org/10.6084/m9.figshare.24551878.v2.

## Ethics statement

The studies involving humans were approved by the institutional Review Board at Abu Dhabi University approved this study under the code number CoHS – 22-05-00018. The studies were conducted in accordance with the local legislation and institutional requirements. The participants provided their written informed consent to participate in this study.

## Author contributions

KI: Conceptualization, Investigation, Methodology, Project administration, Supervision, Validation, Writing – original draft, Writing – review & editing. DR: Validation, Writing – original draft. PS: Data curation, Formal analysis, Software, Validation, Writing – review & editing. CH: Data curation, Formal analysis, Software, Visualization, Writing – review & editing. HS: Writing – review & editing. MB: Investigation, Writing – review & editing. RD: Investigation, Writing – review & editing. SE: Investigation, Writing – review & editing. JS: Investigation, Writing – review & editing. FS: Investigation, Writing – review & editing. MR: Conceptualization, Validation, Writing – review & editing. HH: Conceptualization, Validation, Writing – review & editing. MC: Conceptualization, Funding acquisition, Methodology, Project administration, Supervision, Validation, Writing – original draft.

## Footnotes

^1^ https://terrance.who.int/mediacentre/data/WHODAS/Guidelines/WHODAS%202.0%20Translation%20guidelines.pdf

^2^ https://www.psychometrica.de/effect_size.html
